# On Some Transverse Geometrical Structures of Lifted Foliation to Its Conormal Bundle

**DOI:** 10.1155/2015/218912

**Published:** 2015-03-05

**Authors:** Cristian Ida, Alexandru Oană

**Affiliations:** Department of Mathematics and Computer Science, Transilvania University of Braşov, Street Iuliu Maniu 50, 500091 Braşov, Romania

## Abstract

We consider the lift of a foliation to its conormal bundle and some transverse geometrical structures associated with this foliation are studied. We introduce a good vertical connection on the conormal bundle and, moreover, if the conormal bundle is endowed with a transversal Cartan metric, we obtain that the lifted foliation to its conormal bundle is a Riemannian one. Also, some transversally framed *f*(3, *ε*)-structures of corank 2 on the normal bundle of lifted foliation to its conormal bundle are introduced and an almost (para)contact structure on a transverse Liouville distribution is obtained.

## 1. Introduction and Preliminaries

The study of the lift of transversal Finsler foliations to their normal bundle using the technique of good vertical connection was initiated by Miernowski and Mozgawa [1] where it is proved that the lifted foliation is a Riemannian one. Also, using different methods, some connections between foliations and Lagrangians (or Hamiltonians) in order to recover Riemannian foliations are investigated in the recent papers [2–5]. Our aim in this paper is to extend the study from [1] for the case of lifted foliation to its conormal bundle. In this sense we introduce a good vertical connection on the conormal bundle and we give an application of it in order to obtain that the lifted foliation is a Riemannian one in the case when the conormal bundle is endowed with a transversal Cartan metric. Moreover, in this case, some transversally framed *f*(3, *ε*)-structures and an almost (para)contact structure associated with lifted foliation are investigated.

The methods used here are similarly and closely related to those used in [1, 6] for the case of transversal Finsler foliations.

Let us consider *M* an (*n* + *m*)-dimensional manifold which will be assumed to be connected and orientable.

**Definition 1 .**

A codimension *n* foliation ℱ on *M* is defined by a foliated cocycle {*U*_*i*_, *φ*_*i*_, *f*_*i*,*j*_} such that

(i) {*U*_*i*_}, *i* ∈ *I*, is an open covering of *M*;
(ii) for every *i* ∈ *I*, *φ*_*i*_ : *U*_*i*_ → *N* are submersions, where *N* is an *n*-dimensional manifold, called transversal manifold;
(iii) the maps *f*_*i*,*j*_ : *φ*_*i*_(*U*_*i*_∩*U*_*j*_) → *φ*_*j*_(*U*_*i*_∩*U*_*j*_) satisfy
  (1) $$\begin{array}{c} \varphi_{j}=f_{i,j}\circ \varphi_{i} \end{array}$$
 for every (*i*, *j*) ∈ *I* × *I* such that *U*_*i*_∩*U*_*j*_ ≠ *∅*.

Every fibre of *φ*_*i*_ is called a plaque of the foliation. Condition (1) says that on the intersection *U*_*i*_∩*U*_*j*_ the plaques defined, respectively, by *φ*_*i*_ and *φ*_*j*_ coincide. The manifold *M* is decomposed into a family of disjoint immersed connected submanifolds of dimension *m*; each of these submanifolds is called a leaf of ℱ.

By *T*ℱ we denote the tangent bundle to ℱ and Γ(ℱ) is the space of its global sections, that is, vector fields tangent to ℱ, and by *Q*ℱ = *TM*/*T*ℱ we denote the normal bundle of ℱ.

In this paper, a system of local coordinates adapted to the foliation ℱ means coordinates (*x* ^1^,…, *x* ^*n*^, *y* ^1^,…, *y* ^*m*^) on an open subset *U* on which the foliation is trivial and defined by the equations *dx* ^*a*^ = 0, *a* = 1,…, *n*.

We notice that the total spaces of the conormal bundle *Q* ^*^ℱ of ℱ carry a natural foliation $\tilde{F}$ of codimension 2*n* such that the leaves of $\tilde{F}$ are covering spaces of the leaves of ℱ, and it is called the natural lift of ℱ to its conormal bundle *Q* ^*^ℱ.

If we denote by {*dx* ^*a*^}, *a* = 1,…, *n*, the corresponding local coframe on *Q* ^*^ℱ, then we can induce a chart (*x* ^*a*^, *p*_*a*_, *y* ^*u*^) on *Q* ^*^ℱ where *p* = *p*_*a*_ *dx* ^*a*^ ∈ Γ(*Q* ^*^ℱ), and the system of equations *x* ^*a*^ = const., *p*_*a*_ = const. defines the foliation $\tilde{F}$.

Let $Q\tilde{F}=T(Q^{*}F)/T\tilde{F}$ be the normal bundle of the foliated manifold $\left(Q^{*}F,\tilde{F}\right)$. The vectors {∂/∂*x* ^*a*^, ∂/∂*p*_*a*_}, *a* = 1,…, *n*, form a natural frame of $Q\tilde{F}$ at the point (*x* ^*a*^, *p*_*a*_, *y* ^*u*^) ∈ *Q* ^*^ℱ. The canonical projection *π* : *Q* ^*^ℱ → *M* given by *π*(*x* ^*a*^, *p*_*a*_, *y* ^*u*^) = (*x* ^*a*^, *y* ^*u*^) induces another projection *π*_∗_ : *T*(*Q* ^*^ℱ) → *TM* which maps the vectors tangent to $\tilde{F}$ in the vectors tangent to ℱ. Thus, *π*_∗_ induces a mapping ${\tilde{\pi }}_{*}:Q\tilde{F}\to QF$ and is denoted by $V(Q^{*}F)=\text{ker}{\tilde{\pi }}_{*}$ which is a vertical bundle spanned by the vectors {∂/∂*p*_*a*_}, *a* = 1,…, *n*.

**Lemma 2 .**

Let *o* : *M* → *Q* ^*^ℱ be the zero section of the conormal bundle *Q* ^*^ℱ. Then the set *o*(*M*) is saturated on *Q* ^*^ℱ with foliation $\tilde{F}$.

## 2. Good Vertical Connection on $\left(Q^{*}F,\tilde{F}\right)$

The purpose of this section is to define a linear connection $\nabla :X(V(Q^{*}F))\to X({\text{T}}^{*}({\tilde{Q}}^{*}F)⊗V(Q^{*}F))$ related to considered foliated structure, where ${\tilde{Q}}^{*}F=Q^{*}F-o(M)$. Since we have the foliated manifold $\left({\tilde{Q}}^{*}F,\tilde{F}\right)$, we are looking for a Bott connection such that for any vector field *X* tangent to $\tilde{F}$ and any transversal vector field *Y* we have

(2) $$\begin{array}{c} \nabla_{X}Y=p_{Q\tilde{F}}\left(\left[X,\tilde{Y}\right]\right), \end{array}$$

where $p_{Q\tilde{F}}:T(Q^{*}F)\to Q\tilde{F}$ is the canonical projection and $p_{Q\tilde{F}}(\tilde{Y})=Y$.

Let us consider now the $\tilde{F}$-transversal Hamilton-Liouville vector field defined by *C* ^*^ : *Q* ^*^ℱ → *V*(*Q* ^*^ℱ), *C* ^*^(*x* ^*a*^, *p*_*a*_, *y* ^*u*^) = *p*_*a*_(∂/∂*p*_*a*_). It can be checked that this definition is well posed. From the definition of the Bott connection, the following lemma holds.

**Lemma 3 .**

Let $\nabla :X(V(Q^{*}F))\to X(T^{*}({\tilde{Q}}^{*}F)⊗V(Q^{*}F))$ be a Bott connection. Then ∇_*X*_ *C* ^*^ = 0 for every vector field tangent to $\tilde{F}$.

Now, consider the local frame {∂/∂*x* ^*a*^, ∂/∂*p*_*a*_, ∂/∂*y* ^*u*^} of *T*(*Q* ^*^ℱ) and recall that the vectors {∂/∂*p*_*a*_} form the basis of *V*(*Q* ^*^ℱ). With these settings we put

(3) $$\begin{array}{c} \nabla_{\partial /\partial x^{a}}\frac{\partial }{\partial p_{b}}=\Gamma_{ac}^{b}\frac{\partial }{\partial p_{c}},\;\;\nabla_{\partial /\partial p_{a}}\frac{\partial }{\partial p_{b}}=\Gamma_{c}^{ab}\frac{\partial }{\partial p_{c}},\\ \nabla_{\partial /\partial y^{u}}\frac{\partial }{\partial p_{b}}=\Gamma_{uc}^{b}\frac{\partial }{\partial p_{c}}. \end{array}$$

From the above formulas it follows that

(4) $$\begin{array}{c} \Gamma_{uc}^{b}=0,\;\;\nabla_{\partial /\partial p_{a}}C^{*}=\left(\delta_{c}^{a}+p_{b}\Gamma_{c}^{ab}\right)\frac{\partial }{\partial p_{c}}. \end{array}$$

The Bott connection ∇ allows us to define a mapping

(5) $$\begin{array}{c} L:X\left(Q\tilde{F}\right)⟶X\left(V\left(Q^{*}F\right)\right),\;\;L\left(X\right)=\nabla_{\tilde{X}}C^{*}, \end{array}$$

where $p_{Q\tilde{F}}(\tilde{X})=X$. If we denote by Λ the restriction of the linear mapping *L* to the bundle *V*(*Q* ^*^ℱ), then we can state the following.

**Definition 4 .**

The Bott connection ∇ is said to be a good vertical connection if Λ : *V*(*Q* ^*^ℱ) → *V*(*Q* ^*^ℱ) is a bundle isomorphism.

Observe that ∇ is a good vertical connection if and only if the matrix *δ*_*c*_^*a*^ + *p*_*b*_Γ_*c*_^*ab*^ is nondegenerated. If we put *H*(*Q* ^*^ℱ) = ker*L*, then we can split the bundle $Q\tilde{F}$ into direct sum:

(6) $$\begin{array}{c} Q\tilde{F}=H\left(Q^{*}F\right)⊕V\left(Q^{*}F\right). \end{array}$$

The coefficients of the mapping *L* in the basis {∂/∂*x* ^*a*^, ∂/∂*p*_*a*_} of $Q\tilde{F}$ are

(7) $$\begin{array}{c} L\left(\frac{\partial }{\partial x^{a}}\right)=p_{b}\Gamma_{ac}^{b}\frac{\partial }{\partial p_{c}},\\ L\left(\frac{\partial }{\partial p_{a}}\right)=\left(\delta_{c}^{a}+p_{b}\Gamma_{c}^{ab}\right)\frac{\partial }{\partial p_{c}}=L_{c}^{a}\frac{\partial }{\partial p_{c}}. \end{array}$$

It is easy to check that the vectors *δ*/*δx* ^*a*^ = ∂/∂*x* ^*a*^ + *N*_*ab*_(∂/∂*p*_*b*_), where *N*_*ab*_ = −(*L* ^−1^)_*b*_^*c*^ *p*_*d*_Γ_*ac*_^*d*^, form a basis of ker*L*. In the sequel we will use the basis {*δ*/*δx* ^*a*^, ∂/∂*p*_*a*_}, called adapted, as well as its dual {*dx* ^*a*^, *δp*_*a*_ = *dp*_*a*_ − *N*_*ab*_ *dx* ^*b*^}. Using this coframe we can define the local connection forms by

(8) $$\begin{array}{c} \nabla \frac{\partial }{\partial p_{b}}=\omega_{a}^{b}⊗\frac{\partial }{\partial p_{a}}, \end{array}$$

where

(9) ωab=Γcabdxc+Γabcdpc=Γcab+ΓabdNdcdxc+Γabcδpcωab=Hcabdxc+Γabcδpc.

Notice that *H*_*ca*_^*b*^ *p*_*b*_ = *N*_*ca*_. The formula *θ*(∂/∂*p*_*a*_) = *δ*/*δx* ^*a*^ defines a linear mapping *θ* : *V*(*Q* ^*^ℱ) → *H*(*Q* ^*^ℱ). This mapping allows us to extend the connection ∇ to the horizontal bundle *H*(*Q* ^*^ℱ) by

(10) $$\begin{array}{c} \nabla_{X}Y=\theta \left(\nabla_{X}\theta^{-1}\left(Y\right)\right), \end{array}$$

where *Y* ∈ Γ(*H*(*Q* ^*^ℱ)), *X* ∈ Γ(*T*(*Q* ^*^ℱ)). In this way we construct a linear connection in $Q\tilde{F}$:

(11) $$\begin{array}{c} \nabla_{X}Y=\nabla_{X}\left(v\left(Y\right)\right)+\nabla_{X}\left(Y-v\left(Y\right)\right), \end{array}$$

where $Y\in \Gamma (Q\tilde{F})$, *X* ∈ Γ(*T*(*Q* ^*^ℱ)) and $v:Q\tilde{F}\to V(Q^{*}F)$ is the vertical projection from decomposition (6). In particular we have

(12) $$\begin{array}{c} \nabla \frac{\delta }{\delta x^{a}}=\omega_{a}^{b}⊗\frac{\delta }{\delta x^{b}}, \end{array}$$

where *ω*_*a*_^*b*^ is given in (9).

If $\varphi \in \Gamma \left(Q^{*}\tilde{F}⊗Q\tilde{F}\right)$ is a 1-form with values in $Q\tilde{F}$, locally given by

(13) $$\begin{array}{c} \varphi =\varphi^{a}⊗\frac{\delta }{\delta x^{a}}+\varphi_{b}⊗\frac{\partial }{\partial p_{b}}, \end{array}$$

then, following [1, 7], we can define an exterior differential *Dφ* by putting

(14) $$\begin{array}{c} D\varphi =\left(d\varphi^{a}-\varphi^{a}\wedge \omega_{a}^{c}\right)⊗\frac{\delta }{\delta x^{a}}+\left(d\varphi_{b}-\varphi_{b}\wedge \omega_{c}^{b}\right)⊗\frac{\partial }{\partial p_{c}}. \end{array}$$

A straightforward calculus shows that the above formula is well defined.

The bundle $Q^{*}\tilde{F}⊗Q\tilde{F}$ admits a natural section *η* given by

(15) $$\begin{array}{c} \eta =dx^{a}⊗\frac{\partial }{\partial x^{a}}+dp_{b}⊗\frac{\partial }{\partial p_{b}}=dx^{a}⊗\frac{\delta }{\delta x^{a}}+\delta p_{b}⊗\frac{\partial }{\partial p_{b}}. \end{array}$$

It is clear that the form *η* is well defined.

**Definition 5 .**

The form *ζ* = *Dη* is called the torsion form of the connection ∇.

Locally the form *ζ* can be expressed as follows:

(16) Dη=−dxa∧ωac⊗δδxc+dδpb−δpc∧ωbc⊗∂∂pbDη=ζc⊗δδxc+ζb⊗∂∂pb,

where

(17) $$\begin{array}{c} \zeta^{c}=\frac{1}{2}\left(H_{ea}^{c}-H_{ae}^{c}\right)dx^{a}\wedge dx^{e}-\Gamma_{a}^{ce}dx^{a}\wedge \delta p_{e},\\ \zeta_{b}=-dN_{cb}\wedge dx^{c}-H_{eb}^{a}\delta p_{a}\wedge dx^{e}-\Gamma_{b}^{ae}\delta p_{a}\wedge \delta p_{e}. \end{array}$$

## 3. Transversal Cartan Metrics on *Q* ^*^ℱ and Riemannian Foliations

As in the case of transversal Finsler metrics on the normal bundle of a foliation, [1, 3], a transversal Cartan metric on *Q* ^*^ℱ is a basic function (with respect to the lifted foliation $\tilde{F}$) *K* : *Q* ^*^ℱ → [0, *∞*) which has the following properties:

- *K* is *C* ^*∞*^ on ${\tilde{Q}}^{*}F$;
- *K*(*x*, *λp*) = *λK*(*x*, *p*) for all *λ* > 0;
- the *n* × *n* matrix (*g* ^*ab*^), where *g* ^*ab*^ = (1/2)(∂^2^ *K* ^2^/∂*p*_*a*_∂*p*_*a*_), is positive definite at all points of ${\tilde{Q}}^{*}F$.

Also *K*(*x*, *p*) > 0, whenever *p* ≠ 0. As usual, [8], the properties of *K* imply that

(18) $$\begin{array}{c} p^{a}=g^{ab}p_{b},\;\;p_{a}=g_{ab}p^{b},\;\;K^{2}=g^{ab}p_{a}p_{b}=p_{a}p^{a},\\ C^{abc}p_{c}=C^{acb}p_{c}=C^{cab}p_{c}=0, \end{array}$$

where (*g*_*ab*_) is the inverse matrix of (*g* ^*ba*^) and we have put *p* ^*a*^ = (1/2)(∂*K* ^2^/∂*p*_*a*_), *C* ^*abc*^ = −(1/4)(∂^3^ *K* ^2^/∂*p*_*a*_∂*p*_*b*_∂*p*_*c*_).

Also, *g* ^*ab*^ determines a metric structure on *V*(*Q* ^*^ℱ) by setting

(19) $$\begin{array}{c} G^{v}\left(X,Y\right)=g^{ab}\left(x,p\right)X_{a}\left(x,y,p\right)Y_{b}\left(x,y,p\right), \end{array}$$

for every *X* = *X*_*a*_(*x*, *y*, *p*)(∂/∂*p*_*a*_) and *Y* = *Y*_*b*_(*x*, *y*, *p*)(∂/∂*p*_*b*_) ∈ Γ(*V*(*Q* ^*^ℱ)).

Similar reasons as for transversal Finsler foliations (see Theorem 3.1 from [1]) lead to the following result.

**Theorem 6 .**

Let *K* : *Q* ^*^ℱ → [0, *∞*) be a transversal Cartan metric and let *G* ^*v*^ be the Riemannian metric on *V*(*Q* ^*^ℱ) induced by *K* as in (19). Then there exists exactly one Bott vertical connection $\nabla :X(V(Q^{*}F))\to X(T^{*}({\tilde{Q}}^{*}F)⊗V(Q^{*}F))$ such that

(i) ∇ is a good vertical connection;
(ii) if *X*, *Y* ∈ Γ(*V*(*Q* ^*^ℱ)) and $Z\in \Gamma (T({\tilde{Q}}^{*}F))$, then
  (20) $$\begin{array}{c} {ZG}^{v}\left(X,Y\right)=G^{v}\left(\nabla_{Z}X,Y\right)+G^{v}\left(X,\nabla_{Z}Y\right); \end{array}$$
(iii) *ζ*(*X*, *Y*) = 0 for every *X*, *Y* ∈ Γ(*V*(*Q* ^*^ℱ));
(iv) *ζ*(*X*, *Y*) ∈ Γ(*V*(*Q* ^*^ℱ)) for every *X*, *Y* ∈ Γ(*H*(*Q* ^*^ℱ)).

Also, the isomorphism *θ* does not depend on the coordinates along the leaves of $\tilde{F}$; so the Riemannian metric in $Q\tilde{F}$ defined by *G* = *G* ^*h*^ + *G* ^*v*^, where *G* ^*h*^(*X*, *Y*) = *G* ^*v*^(*θ* ^−1^(*X*), *θ* ^−1^(*Y*)) for every *X*, *Y* ∈ Γ(*H*(*Q* ^*^ℱ)) and *G*(*X*, *Y*) = 0 for every *X* ∈ Γ(*H*(*Q* ^*^ℱ)) and *Y* ∈ Γ(*V*(*Q* ^*^ℱ)), is a transversal Riemannian metric for the lifted foliation $\tilde{F}$ to the conormal bundle *Q* ^*^ℱ of ℱ. Hence, we can consider the following.

**Theorem 7 .**

If the conormal bundle of foliation ℱ is endowed with a transversal Cartan metric, then the lifted foliation $\tilde{F}$ to the conormal bundle *Q* ^*^ℱ is Riemannian.

## 4. Transversally Framed *f*(3, *ε*)-Structures on $\left(Q^{*}F,\tilde{F}\right)$

The study of structures on manifolds defined by a tensor field satisfying *f* ^3^ ± *f* = 0 has the origin in a paper by Yano [9]. Later on, these structures have been generically called *f*-structures. On the tangent manifold of a Finsler space, the notion of framed *f*(3,1)-structure was defined and studied by Anastasiei in [10] and on the cotangent bundle of a Cartan space the study is continued in [11, 12]. Taking into account that the conormal bundle *Q* ^*^ℱ has a local model of a cotangent manifold, in this section we extend the study concerning *f*-structures in our context.

Let *ε* = ±1. A framed *f*(3, *ε*)-structure of corank *s* on a (2*n* + *s*)-dimensional manifold *N* is a natural generalization of an almost contact structure on *N* (for *ε* = 1) and of an almost paracontact structure on *N* (for *ε* = −1), respectively, and it is a triplet (*f*, (*ξ*_*i*_), (*ω* ^*i*^)), *i* = 1,…, *s*, where *f* is a tensor field of type (1,1), (*ξ*_*i*_) are vector fields, and (*ω* ^*i*^) are 1-forms on *N* such that

(21) $$\begin{array}{c} \omega^{i}\left(\xi_{j}\right)=\delta_{j}^{i},\;\;f\left(\xi_{i}\right)=0,\;\;\omega^{i}\circ f=0,\\ f^{2}=-\varepsilon \left(I-\underset{i}{\sum }\omega^{i}⊗\xi_{i}\right), \end{array}$$

where *I* denotes the Kronecker tensor field on *N*. The name of *f*(3, *ε*)-structure was suggested by the identity *f* ^3^ + *εf* = 0. For an account of such kind of structures, we refer to [13].

Let us consider now that *Q* ^*^ℱ is endowed with a transversal Cartan metric *K*. The linear operator *ϕ* given in the local adapted basis of $Q\tilde{F}$ by

(22) $$\begin{array}{c} \phi \left(\frac{\delta }{\delta x^{a}}\right)=-\varepsilon g_{ab}\frac{\partial }{\partial p_{b}},\;\;\phi \left(\frac{\partial }{\partial p_{a}}\right)=g^{ab}\frac{\delta }{\delta x^{b}} \end{array}$$

defines an almost complex structure on $Q\tilde{F}$ for *ε* = 1 and an almost paracomplex structure on $Q\tilde{F}$ for *ε* = −1, respectively. We also have

(23) $$\begin{array}{c} G\left(\phi \left(X\right),\phi \left(Y\right)\right)=G\left(X,Y\right),\;\forall X,Y\in \Gamma \left(Q\tilde{F}\right). \end{array}$$

Let us put *ξ*_1_ = (*p* ^*a*^/*K*)(*δ*/*δx* ^*a*^) and *ξ*_2_ = (*p*_*a*_/*K*)(∂/∂*p*_*a*_) = (1/*K*)*C* ^*^. Thus, we have two global transverse vector fields on $\left(Q^{*}F,\tilde{F}\right)$ which are linearly independent. The first is transversally horizontal and the second one is transversally vertical.

From the definition of *ϕ* it follows

(24) $$\begin{array}{c} \phi \left(\xi_{1}\right)=-\varepsilon \xi_{2},\;\;\phi \left(\xi_{2}\right)=\xi_{1}. \end{array}$$

Now, if we consider the dual transverse 1-forms of *ξ*_1_ and *ξ*_2_, respectively, locally given by *ω* ^1^ = (*p*_*a*_/*K*)*dx* ^*a*^ and *ω* ^2^ = (*p* ^*a*^/*K*)*δp*_*a*_, then we easily check that

(25) ω1∘ϕ=ω2,  ω2∘ϕ=−εω1,ω1X=GX,ξ1, ω2X=GX,ξ2, ∀X∈ΓQF~.

Next, using *ϕ*, *ξ*_*i*_, and *ω* ^*i*^, *i* ∈ {1,2}, we construct the transverse tensor field *f* of type (1,1) on $\left(Q^{*}F,\tilde{F}\right)$ by putting

(26) $$\begin{array}{c} f\left(X\right)=\phi \left(X\right)-\omega^{2}\left(X\right)\xi_{1}+\varepsilon \omega^{1}\left(X\right)\xi_{2},\;X\in \Gamma \left(Q\tilde{F}\right). \end{array}$$

Using a similar argument as in [6, 10–12] by direct calculus, we obtain the following.

**Theorem 8 .**

The triple (*f*, (*ξ*_*i*_), (*ω* ^*i*^)), *i* ∈ {1,2}, provides some transversally framed *f*(3, *ε*)-structures of corank 2 on $\left(Q^{*}F,\tilde{F}\right)$; that is, the following hold:

- *ω* ^*i*^(*ξ*_*j*_) = *δ*_*j*_^*i*^, *f*(*ξ*_*i*_) = 0, *ω* ^*i*^∘*f* = 0;
- *f* ^2^ = −*ε*(*I* − *ω* ^1^ ⊗ *ξ*_1_ − *ω* ^2^ ⊗ *ξ*_2_);
- *f* is of rank 2*n* − 2 and *f* ^3^ + *εf* = 0.

**Theorem 9 .**

The transversal Riemannian metric *G* on $\left(Q^{*}F,\tilde{F}\right)$ satisfies

(27) GfX,fY=GX,Y−ω1Xω1YGfX,fY=−ω2Xω2Y, X,Y∈ΓQF~.

For *ε* = 1 we put

(28) $$\begin{array}{c} \Phi \left(X,Y\right)=G\left(f\left(X\right),Y\right),\;X,Y\in \Gamma \left(Q\tilde{F}\right). \end{array}$$

By using Theorems 8 and 9, we obtain

(29) $$\begin{array}{c} \Phi \left(X,Y\right)=-\Phi \left(Y,X\right),\;X,Y\in \Gamma \left(Q\tilde{F}\right). \end{array}$$

Thus, Φ is a transverse 2-form on $\left(Q^{*}F,\tilde{F}\right)$. It is degenerate with null space span⁡{*ξ*_1_, *ξ*_2_}.

Also, using the calculus in local coordinates, we easily obtain

(30) $$\begin{array}{c} \Phi \left(\frac{\delta }{\delta x^{a}},\frac{\delta }{\delta x^{b}}\right)=0,\;\;\Phi \left(\frac{\delta }{\delta x^{a}},\frac{\partial }{\partial p_{b}}\right)=-\delta_{a}^{b}+\frac{p_{a}p^{b}}{K^{2}},\\ \Phi \left(\frac{\partial }{\partial p_{a}},\frac{\partial }{\partial p_{b}}\right)=0. \end{array}$$

On the other hand, we have

(31) dω1δδxa,δδxb=δδxapbK−δδxbpaK,dω1δδxa,∂∂pb=1K−δab+papbK2,dω1∂∂pa,∂∂pb=0,

where in the second relation we have used *p* ^*a*^ = *K*(∂*K*/∂*p*_*a*_) which follows from the homogeneity conditions (18) of *K*. Comparing now Φ with *dω* ^1^ we obtain

(32) $$\begin{array}{c} \frac{1}{K}\Phi =d\omega^{1}+\Psi , \end{array}$$

where Ψ = ((*δ*/*δx* ^*b*^)(*p*_*a*_/*K*) − (*δ*/*δx* ^*a*^)(*p*_*b*_/*K*))*dx* ^*a*^∧*dx* ^*b*^. Thus, (1/*K*)Φ is transversally closed if and only if Ψ is transversally closed. Concluding, (1/*K*)Φ is in general an almost transversally presymplectic structure on $\left(Q^{*}F,\tilde{F}\right)$.

Similarly, for *ε* = −1, we can put

(33) $$\begin{array}{c} H\left(X,Y\right)=G\left(f\left(X\right),Y\right),\;X,Y\in \Gamma \left(Q\tilde{F}\right). \end{array}$$

We have the following.

**Theorem 10 .**

The mapping *H* is a symmetric bilinear form on $\left(Q^{*}F,\tilde{F}\right)$ and the annihilator of *H* is ker*f*.

**Proof**

The symmetry and bilinearity are obvious. Also, the null space of *H* is

(34) X∈ΓQF~ ∣ HX,Y=0,  ∀Y∈ΓQF~ =X∈ΓQF~ ∣ GfX,Y=0=kerf

which end the proof.

Locally, we obtain

(35) $$\begin{array}{c} H=\left(g_{ab}-\frac{p_{a}p_{b}}{K^{2}}\right)dx^{a}⊗dx^{b}-\left(g^{ab}-\frac{p^{a}p^{b}}{K^{2}}\right)\delta p_{a}⊗\delta p_{b} \end{array}$$

with det⁡(*g*_*ab*_ − (*p*_*a*_ *p*_*b*_/*K* ^2^)) = 0, since (*g*_*ab*_ − (*p*_*a*_ *p*_*b*_/*K* ^2^))*p* ^*b*^ = *p*_*a*_ − *p*_*a*_ = 0, and similarly det⁡(*g* ^*ab*^ − (*p* ^*a*^ *p* ^*b*^/*K* ^2^)) = 0, since (*g* ^*ab*^ − (*p* ^*a*^ *p* ^*b*^/*K* ^2^))*p*_*b*_ = *p* ^*a*^ − *p* ^*a*^ = 0.

**Remark 11 .**

The map *H* is a transversally singular pseudo-Riemannian metric on $\left(Q^{*}F,\tilde{F}\right)$.

## 5. An Almost (Para)Contact Structure on Transverse Liouville Distribution of $\left(Q^{*}F,\tilde{F}\right)$

Denote by {*ξ*_2_} the line vector bundle over *Q* ^*^ℱ spanned by *ξ*_2_ and we define the transverse vertical Liouville distribution as the complementary orthogonal distribution *S*(*Q* ^*^ℱ) to {*ξ*_2_} in *V*(*Q* ^*^ℱ) with respect to *G* ^*v*^; namely, *V*(*Q* ^*^ℱ) = *S*(*Q* ^*^ℱ)⊕{*ξ*_2_}. Hence, *S*(*Q* ^*^ℱ) is defined by *α* : = *ω* ^2^|_*V*(Q^*^ℱ)_; that is,

(36) $$\begin{array}{c} \Gamma \left(S\left(Q^{*}F\right)\right)=\left\{X\in \Gamma \left(V\left(Q^{*}F\right)\right);\;\;\alpha \left(X\right)=0\right\}. \end{array}$$

Thus, any transverse vertical vector field *X* ∈ Γ(*V*(*Q* ^*^ℱ)) can be expressed as

(37) $$\begin{array}{c} X=PX+\alpha \left(X\right)\xi_{2}, \end{array}$$

where *P* is the projection morphism of *V*(*Q* ^*^ℱ) on *S*(*Q* ^*^ℱ). By direct calculations, one gets the following.

**Proposition 12 .**

For any transverse vertical vector fields *X*, *Y* ∈ Γ(*V*(*Q* ^*^ℱ)), one has

(38) $$\begin{array}{c} G^{v}\left(X,PY\right)=G^{v}\left(PX,PY\right)=G^{v}\left(X,Y\right)-\alpha \left(X\right)\alpha \left(Y\right). \end{array}$$

**Theorem 13 .**

The transverse vertical Liouville distribution *S*(*Q* ^*^ℱ) is integrable.

**Proof**

The proof follows using an argument similar to Theorem 3.1 [14] (see also Theorem 2.1 [6] or Theorem 4 [15]).

In the following, we will consider the transverse Liouville distribution of $\left(Q^{*}F,\tilde{F}\right)$ as the complementary orthogonal distribution $\bar{S}(Q^{*}F)$ to {*ξ*_2_} in $Q\tilde{F}$ with respect to *G*; that is, $\bar{S}(Q^{*}F)=H(Q^{*}F)⊕S(Q^{*}F)$.

Let us restrict to $\bar{S}(Q^{*}F)$ all the geometrical structures introduced in Section 4 for all $Q\tilde{F}$. We indicate this by overlines. Hence, we have

(i) $\bar{\xi_{1}}=\xi_{1}$ since *ξ*_1_ lies in $\bar{S}(Q^{*}F)$;
(ii) $\bar{\omega^{2}}=0$ since *ω* ^2^(*X*) = *G*(*X*, *ξ*_2_) = 0 for every transverse vector field $X\in \bar{S}(Q^{*}F)$;
(iii) $\bar{G}=G{|}_{\bar{S}(Q^{*}F)}$;
(iv) $\bar{f}(X)=\bar{\phi }(X)+\varepsilon \bar{\omega^{1}}(X)⊗\xi_{2}$ is an endomorphism of $\bar{S}(Q^{*}F)$ since
  (39) Gf¯X,ξ2=Gϕ¯X,ξ2+εω1¯XGξ2,ξ2Gf¯X,ξ2=ω2(ϕ¯(X))+εω1¯(X)=0.

We denote now $\bar{\xi }=\bar{\xi_{1}}$ and $\bar{\eta }=\bar{\omega^{1}}$.

By Theorem 8, we obtain the following.

**Theorem 14 .**

The triple $\left(\bar{f},\bar{\xi },\bar{\eta }\right)$ provides an almost (para)contact structure on $\bar{S}(Q^{*}F)$; that is,

- ${\bar{f}}^{3}+\varepsilon \bar{f}=0$, $rank\bar{f}=2n-2=(2n-1)-1$;
- $\bar{\eta }(\bar{\xi })=1$, $\bar{f}(\bar{\xi })=0$, $\bar{\eta }\circ \bar{f}=0$;
- ${\bar{f}}^{2}(X)=-\varepsilon (X-\bar{\eta }(X)\bar{\xi })$, for $X\in \bar{S}(Q^{*}F)$.

Also, by Theorem 9 we obtain the following.

**Theorem 15 .**

The transversal Riemannian metric $\bar{G}$ verifies

(40) $$\begin{array}{c} \bar{G}\left(\bar{f}\left(X\right),\bar{f}\left(Y\right)\right)=\bar{G}\left(X,Y\right)-\bar{\eta }\left(X\right)\bar{\eta }\left(Y\right), \end{array}$$

for every transverse vector fields $X,Y\in \bar{S}(Q^{*}F)$.

Concluding, the ensemble $\left(\bar{f},\bar{\xi },\bar{\eta },\bar{G}\right)$ is an almost (para)contact Riemannian structure on $\bar{S}(Q^{*}F)$.

## References

[1] Miernowski A., Mozgawa W. (2006). Lift of the Finsler foliation to its normal bundle. *Differential Geometry and Its Applications*.

[2] Popescu P., Ida C. Foliations compatible with hamiltonians.

[3] Popescu P., Popescu M. (2009). Lagrangians adapted to submersions and foliations. *Differential Geometry and Its Applications*.

[4] Popescu P., Popescu M. (2011). Foliated vector bundles and Riemannian foliations. *Comptes Rendus Mathematique*.

[5] Popescu P., Popescu M. (2011). Lagrangians and Hamiltonian structures and foliations. *Physics AUC*.

[6] Ida C. (2011). Some framed *f*-structures on transversally Finsler foliations. *Annales Universitatis Mariae Curie-Sklodowska, Sectio A: Mathematica*.

[7] Abate M., Patrizio G. (1994). *Finsler Metrics—A Global Approach*.

[8] Miron R., Hrimiuc D., Shimada H., Sabău S. V. (2001). *The Geometry of Hamilton and Lagrange Spaces*.

[9] Yano K. (1963). On a structure defined by a tensor field of type (1,1) satisfying *f*
^3^ + *f* = 0. *Tensor*.

[10] Anastasiei M. (2000). A framed f-structure on tangent bundle of a Finsler space. *Analele Universitatii Bucuresti, Seria Matematica*.

[11] Gîrtu M. (2004). An almost 2-paracontact structure on the cotangent bundle of a Cartan space. *Hacettepe Journal of Mathematics and Statistics*.

[12] Gîrtu M. (2007). A Sasakian structure on the indicatrix bundle of a Cartan space. *Analele Ştiinţifice ale Universitatii “Al.I.Cuza” din Iasi, Tomul LIII*.

[13] Mihai I., Rosca R., Verstraelen L. (1996). *Some Aspects of the Differential Geometry of Vector Fields*.

[14] Bejancu A., Farran H. R. (1999). On the vertical bundle of a pseudo-Finsler manifold. *International Journal of Mathematics and Mathematical Sciences*.

[15] Ida C., Manea A. (2014). A vertical Liouville subfoliation on the cotangent bundle of a Cartan space and some related structures. *International Journal of Geometric Methods in Modern Physics*.

